# The effectiveness of substance use interventions for homeless and vulnerably housed persons:  A systematic review of systematic reviews on supervised consumption facilities, managed alcohol programs, and pharmacological agents for opioid use disorder

**DOI:** 10.1371/journal.pone.0227298

**Published:** 2020-01-16

**Authors:** Olivia Magwood, Ginetta Salvalaggio, Michaela Beder, Claire Kendall, Victoire Kpade, Wahab Daghmach, Gilbert Habonimana, Zack Marshall, Ellen Snyder, Tim O’Shea, Robin Lennox, Helen Hsu, Peter Tugwell, Kevin Pottie

**Affiliations:** 1 C.T. Lamont Primary Health Care Research Centre, Bruyère Research Institute, Ottawa, ON, Canada; 2 Department of Family Medicine, University of Alberta, Edmonton, AB, Canada; 3 St. Michael’s Hospital, University of Toronto Dept of Psychiatry, Toronto, ON, Canada; 4 Departments of Family Medicine & School of Epidemiology and Public Health, University of Ottawa, Ottawa, ON, Canada; 5 Department of Medicine, McGill University, Montreal, QC, Canada; 6 School of Social Work, McGill University, Montreal, QC, Canada; 7 Public Health and Preventative Medicine Residency Program, University of Ottawa, Ottawa, ON, Canada; 8 Department of Medicine, Population Health Research Institute, McMaster University, Hamilton, ON, Canada; 9 Department of Family Medicine, McMaster University, Hamilton, ON, Canada; 10 Department of Medicine, University of Ottawa, Ottawa, ON, Canada; Centre for Addiction and Mental Health, CANADA

## Abstract

**Background:**

Substance use is disproportionately high among people who are homeless or vulnerably housed. We performed a systematic overview of reviews examining the effects of selected harm reduction and pharmacological interventions on the health and social well-being of people who use substances, with a focus on homeless populations.

**Methods and findings:**

We searched MEDLINE, EMBASE, PsycINFO, Joanna Briggs Institute EBP, Cochrane Database of Systematic Reviews and DARE for systematic reviews from inception to August 2019. We conducted a grey literature search and hand searched reference lists. We selected reviews that synthesized evidence on supervised consumption facilities, managed alcohol programs and pharmacological interventions for opioid use disorders. We abstracted data specific to homeless or vulnerably housed populations. We assessed certainty of the evidence using the GRADE approach.

Our search identified 483 citations and 30 systematic reviews met all inclusion criteria, capturing the results from 442 primary studies. This included three reviews on supervised consumption facilities, 24 on pharmacological interventions, and three on managed alcohol programs. Supervised consumption facilities decreased lethal overdoses and other high risk behaviours without any significant harm, and improved access to care. Pharmaceutical interventions reduced mortality, morbidity, and substance use, but the impact on retention in treatment, mental illness and access to care was variable. Managed alcohol programs reduced or stabilized alcohol consumption. Few studies on managed alcohol programs reported deaths.

**Conclusions:**

Substance use is a common chronic condition impacting homeless populations. Supervised consumption facilities reduce overdose and improve access to care, while pharmacological interventions may play a role in reducing harms and addressing other morbidity. High quality evidence on managed alcohol programs is limited.

## Introduction

Homeless and vulnerably housed populations experience disproportionately high rates of substance use [1–3]. While individuals may use substances as a way to cope with adverse living conditions, stress, and the trauma of homelessness [4–6] it is also the case that without stable housing individuals often experience barriers to accessing and following treatment recommendations for substance use disorders [7]. Childhood traumatic experiences, posttraumatic stress disorder and adult exposure to other traumatic experiences can also influence substance use [8]. Despite the substantial unmet care needs of this population, people struggling with both substance use and homelessness experience overlapping barriers to accessing care, including stigma related to care itself [9], and abstinence-based treatment first housing services [10,11]. Together, these barriers increase perceived loss of control over one’s life, contribute to mistrust of the health system, and perpetuate lower access and adherence to care and treatment [12–14].

There is substantial literature demonstrating that people who are homeless benefit from receiving tailored, patient-centred care within interprofessional primary care teams with an integrated approach to community and social services [15–17]. Specifically, harm reduction and pharmacological interventions represent important approaches to facilitate care and treatment of individuals experiencing homelessness and concomitant substance use disorders. At its core, harm reduction is a pragmatic approach that aims to reduce the adverse consequences of substance use and related socio-cultural marginalization [18–20]. Within a harm reduction approach, specific interventions include opioid overdose response with naloxone, supervised consumption facilities, and managed alcohol programs [21]. Harm reduction approaches are particularly relevant for homeless populations given the extreme health inequities and stigma impacting both substance use and homelessness [1,22,23]. The effectiveness of medications for substance use disorders including methadone, buprenorphine, slow-release morphine, diacetylmorphine, and naltrexone have been documented in the general population [24,25], but the evidence specific to homeless populations is yet to be synthesized. This review is one of a series of reviews to study the effectiveness of interventions for the care of homeless, vulnerably housed, and persons with lived homelessness experience. The objective of this review is to assess the effectiveness of specific harm reduction and pharmacological interventions among homeless or vulnerably housed individuals with concomitant substance use disorders.

## Methods

This systematic review adheres to the guidance provided in the Cochrane Handbook for overviews of reviews [26] and is reported according to the Preferred Reporting Items of Systematic Reviews and Meta-Analyses (PRISMA) guidelines [27] (S1 File). Ethical approval was not required for this study.

### Selection of priority interventions

We conducted a Delphi consensus process with 84 experienced healthcare practitioners and 76 persons with lived homelessness experience to prioritize person-centered and clinically meaningful community interventions, outcomes, and marginalized subgroups [28]. Among these, addiction care was highly prioritized. We then scoped literature using Google Scholar and PubMed to determine a list of interventions and terms relating to each of the Delphi priority topic categories. Priority topic working groups were formed to arrive at a consensus and inform the final selection of the interventions to be included in this review. Each working group consisted of medical practitioners, allied health professionals, and community scholars (people with lived experience of homelessness or vulnerable housing). Our working group deliberated the value of systematic reviews and evidence based guidelines on various addiction interventions, giving significant weight to the needs and opinions of persons with lived experience of homelessness. The working group agreed that focusing on safe consumption sites (of drugs and/or alcohol, i.e. SCFs and MAPs) and medications to manage opioid use disorder should be the priority.

### Search strategy and selection criteria

Our initial search of published literature revealed no experimental trials conducted specifically among homeless populations [29]. We therefore followed the GRADE approach and expanded our search strategy to include grey literature and revised our inclusion criteria to incorporate general population and systematic review evidence for the interventions of interest, and published a protocol reflecting these changes on the Cochrane Equity Methods website [30]. We made this decision fully aware that most studies among “general populations” had a large representation of homeless populations in their samples, and we did not identify any substantial reason to believe that the mechanisms of action of our interventions of interest would differ between homeless populations who use substances and the general population of people who use substances.

We included systematic reviews of quantitative or qualitative studies that focused on at least one of the interventions specified in our protocol among homeless or general populations. Any type of comparator was considered eligible (such as usual services, alternative intervention or no intervention). We included all outcomes of interest including: mortality, morbidity, substance use, mental health, access to care, and retention in treatment. We excluded overviews of reviews and narrative literature reviews (See Table 1 for full inclusion and exclusion criteria).

**Table 1:** Inclusion and exclusion criteria.

**Inclusion Criteria**	**Definitions**	
Population	People experiencing homelessness and vulnerable housing, defined as: “An individual who lacks stable, permanent, appropriate housing, or may be without immediate prospect, means and ability of acquiring it. There are four physical living situations involved with homelessness: 1) Unsheltered; 2) Emergency sheltered; 3) Provisionally accommodated and, 4) At risk of homelessness, referring to people who are not homeless, but whose current economic and/or housing situation is precarious or does not meet public health and safety standards” [31]. We defined vulnerable housing as: “someone who lives in one’s own room or apartment but has unstable living arrangements, often resulting in frequent transitions between homelessness and vulnerable housing” [32]. We did not set any restrictions on the timeline of homelessness (e.g. current, lifetime, past year, etc.). If no studies specific to homeless populations were identified for a given intervention, we expanded our inclusion criteria to the general population who use substances.	
Interventions	Supervised consumption facilities	Legally sanctioned facilities where people who use substances can consume pre-obtained substances under supervision [33]. There exist various terminologies for these facilities, including: supervised injection facilities (SIF), supervised consumption sites (SCS), medically supervised injection centres (MCIS), among others.
	Managed alcohol programs	Shelter, medical assistance, social services and the provision of regulated alcohol to help residents cope with severe alcohol use disorder [34]
	Pharmacological interventions for opioid use disorder	Opioid therapy medications including methadone, buprenorphine, diacetylmorphine, levo-α-acetylmethadol (LAAM) and naltrexone.
	Pharmacologic agents for reversal of opioid overdose	Opioid antagonist administered intravenously or intranasally, e.g. naloxone.
Comparison	No intervention, standard intervention, alternative intervention, treatment as usual.	
Outcomes	1. Mortality: All-cause mortality and rates of suicide. 2. Morbidity: Having a disease or a symptom of disease. For example, prevalence or incidence of a communicable or non-communicable disease. Morbidity also refers to medical problems caused by a treatment. 3. Substance use: As measured by the number of days using alcohol or substance, the rate, and frequency of using alcohol or substances, number of days of abstinence from alcohol or substances or physical and mental consequences of using alcohol or substances. 4. Mental health: Any measures assessing psychological status and wellbeing, including but not limited to, psychological distress, self‐reported mental health status, or mental illness symptoms. 5. Access to care: Access to health care means having "the timely use of personal health services to achieve the best health outcomes" [35]. Access to health care consists of four components [36]: • Coverage: facilitates entry into the health care system. Uninsured people are less likely to receive medical care and more likely to have poor health status. • Services: Having a usual source of care is associated with adults receiving recommended screening and prevention services. • Timeliness: ability to provide health care when the need is recognized. • Workforce: capable, qualified, culturally competent providers. 6. Retention in treatment: The length of time clients remain in treatment.	
Study design	Systematic review of quantitative or qualitative studies. Exclude all other study designs and review types.	
**Exclusion Criteria**	**Justifications**	
Reviews that focus on low- and middle-income countries	Due to the variability in access to resources and supports in comparison to that in a high-income country vary greatly. We feel that the settings are different and should be synthesized separately.	
Reviews that focus on Indigenous populations	The analysis of the interventions tailored to this population will be covered by an Indigenous research group	
Reviews which focus on incarcerated populations	Not generalizable to the non-incarcerated homeless population.	
Reviews which exclusively report on interventions for detoxification	Abstinence-based approaches are outside of the scope of this review	

We developed a systematic search using relevant keywords and MeSH terms for relevant published systematic reviews. Keywords included terms such as “opioid-related disorders”, “supervised consumption”, “supervised injection”, “managed alcohol”, “methadone” and “harm reduction” (See S2 File). We searched MEDLINE, EMBASE, PsycINFO, Joanna Briggs Institute EBP, Cochrane Database of Systematic Reviews and the Database of Abstracts of Reviews of Effects (DARE) for systematic reviews from database inception to August 2019. There were no language restrictions. The reference lists for all articles selected for full-text review were manually searched for relevant citations. These were cross-referenced against our original search results and any additional potentially relevant citations were screened. We conducted a grey literature search (see S2 File) and consulted experts in the field for any additional studies.

Results were uploaded to Rayyan reference manager software to facilitate the study selection process [37]. Two review authors independently assessed each study for inclusion by title/abstract, followed by full-text; disagreements were resolved through discussion or a third reviewer.

### Data analysis

We developed a standardized data extraction template which included study design, search parameters, inclusion criteria, number and design of included studies, results and conclusions. We extracted study details specific to homelessness or housing instability status of participants when available. We extracted data in duplicate and resolved disagreements through discussion. We assessed the methodological quality of reviews in duplicate using AMSTAR 2 (See S3 File) [38].

Following guidance from the Cochrane Handbook [39], we assessed primary study overlap within the included systematic reviews using a citation matrix (See S4 File). We synthesized outcome data narratively and organized the presentation of evidence by intervention. A narrative synthesis is defined as a “synthesis of findings from multiple studies that relies primarily on the use of words and text to summarize and explain the findings of the synthesis. Whilst it can involve the manipulation of statistical data, the defining characteristic is that it adopts a textual approach to the process of synthesis to ‘tell the story’ of the findings from the included studies” [40]. Where appropriate, we included existing meta-analyses with pooled effects, and report estimates of effect as reported in the source review. Numerical estimates of association are only reported in instances when these were provided in the source review. We assessed the certainty of the available numerical evidence for critical patient-important outcomes (mortality, morbidity, substance use) using GRADE methodology, considering risk of bias of the primary studies, indirectness, imprecision and inconsistency [26] (See Table 2: GRADE definitions). Where data was unavailable, a GRADE assessment was not done.

**Table 2:** GRADE certainty of evidence and definitions.

Certainty rating	Definition
High	Further research is very unlikely to change our confidence in the estimate of effect
Moderate	Further research is likely to have an important impact on our confidence in the estimate of effect and may change the estimate
Low	Further research is very likely to have an important impact on our confidence in the estimate of effect and is likely to change the estimate
Very low	Any estimate of effect is very uncertain

### Role of the funding source

The funders of the study had no role in study design, data collection, data analysis, data interpretation, or writing of the report. The corresponding author had full access to all of the data in the study and had final responsibility for the decision to submit for publication.

## Results

**Fig 1:**
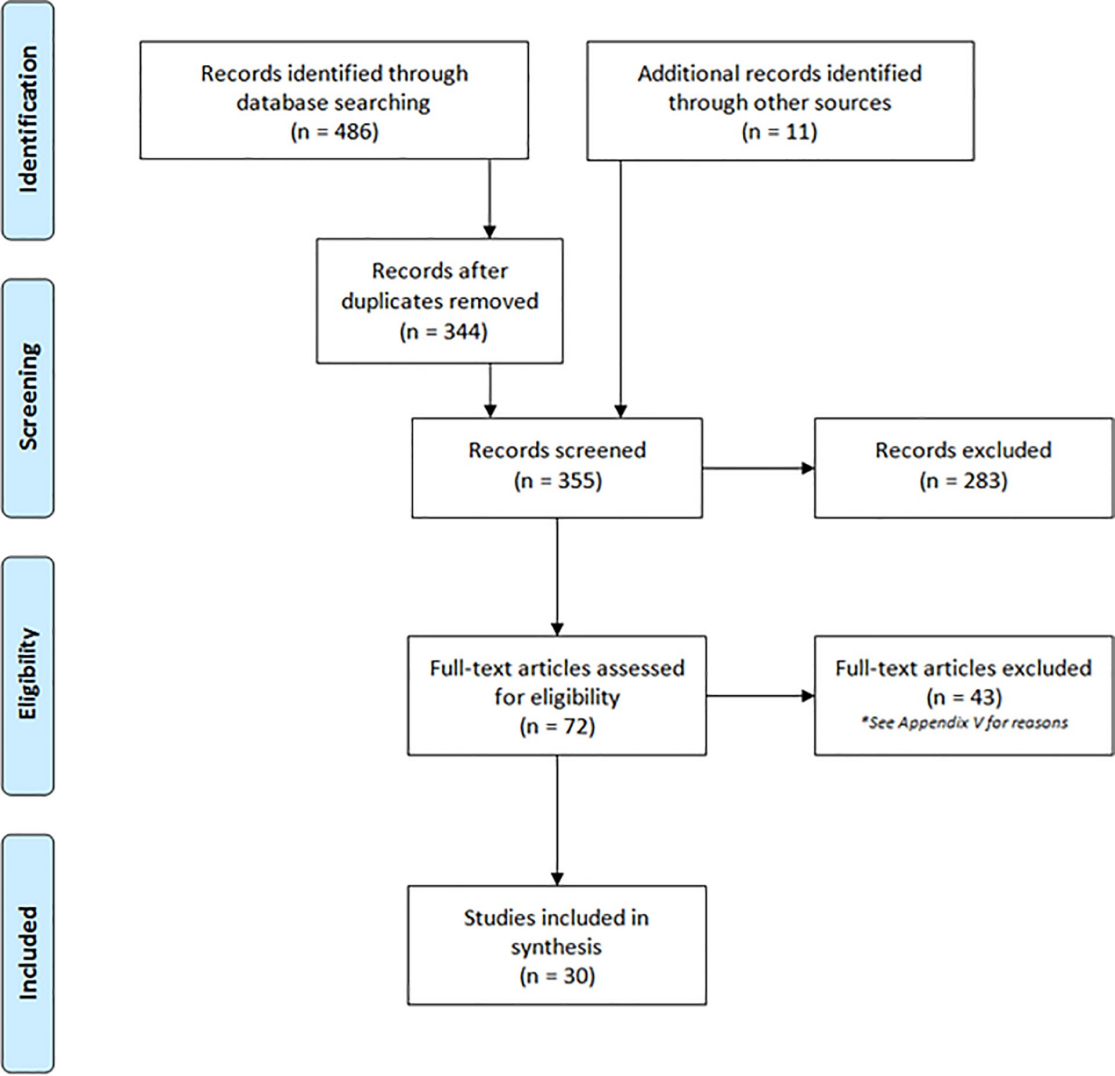
PRISMA flow diagram. Original search ran on July 05, 2019. Search was updated on August 21, 2019.

Our systematic search identified 486 citations. We identified an additional 11 articles through grey literature and reference searching. After removal of duplicates, we screened 355 unique articles on title and abstract, excluded 283 articles and screened 72 articles by full text. Of these, we excluded 42 articles (See S5 File: Table of excluded studies) and included 30 systematic reviews in our final synthesis (See Fig 1: PRISMA).

Of the 30 included systematic reviews, 24 reviews which included 352 unique primary studies reported on pharmacological interventions for opioid use disorder [41–64]. Two systematic reviews which included 90 unique observational studies and one qualitative meta-synthesis reported on supervised consumption facilities (SCFs) [65–67]. One Cochrane review with no included studies [68] and two grey literature reviews reported on 51 studies of managed alcohol programs (MAPs) [69,70].

Fifty-eight percent (14/24) of systematic reviews on pharmacological interventions included studies which reported housing instability or homelessness [41,42,44,46,50,51,53,55–60,64]. All systematic reviews on SCFs included observational studies reporting the rate of homelessness ranging from 10% to as high as 75% among SCF clients, the majority of studies were from Sydney, Australia and Vancouver, Canada [65–67]. Evidence identified on MAPs was specific to homeless populations [69,70]. See Table 3 for additional study characteristics.

**Table 3:** Characteristics of included studies.

Review	Quality (AMSTAR II)	Objective	Included Studies	Population	Outcomes					
					Mortality	Morbidity	Substance use	Mental Health	Access to care	Retention in treatment
Supervised consumption facilities										
Kennedy 2017 [65]	CRITICALLY LOW	To systematically review the literature on public health and public order outcomes of supervised consumption facilities	n = 47	People who use or inject drugs and the broader community in which SCFs were located	The majority of studies reporting on this outcome suggested a protective effect of SCF on overdose deaths	There was a decline in opioid poisoning emergency department presentations as well as ambulance attendances. One study found that SCF clients were more likely to experience an overdose within the SCF	The majority of studies reporting on this outcome showed that SCF participants were less likely to report syringe sharing behaviours as well as unsafe injection behaviours.	N/A	One study found that being advised to seek treatment by SCF staff was associated with higher rate of receiving treatment. Referral by SCF staff was associated with higher access to EDs and shorter durations of hospitalization	Four studies reported better use and uptake of addiction treatment among SCF users.
McNeil & Small 2014 [67]	NOT APPLICABLE	To develop a comprehensive understanding of safer environment interventions (supervised injection facilities, syringe exchange programs and peer interventions) informed by the experiences of people who inject drugs.	N = 21	People who inject drugs	N/A	SIFs produced social and physical settings that enable safer injection practices and reductions in risk behaviour.	N/A	N/A	Mediates access to ancillary services (e.g. food and shelter) and fostered access to broader health supports. These interventions were participants’ primary source of medical care.	N/A
Potier 2014 [66]	CRITICALLY LOW	To systematically collect and synthesize available evidence on supervised injection services benefits and harms	n = 75	Supervised injection services users	The majority of studies reported lower overdose-induced mortalities	Two studies reported lower number of calls for ambulances related to overdose among SIS users	The majority of studies showed that SIS was associated with lower rate of syringe sharing and reuse as well as public-space injection	N/A	All studies on this outcome reported a global increase in referral to addiction treatment centres and detoxification programs	N/A
Managed alcohol programs										
Ezzard 2015 [69]	CRITICALLY LOW	To address the gap of evidence on the feasibility and acceptability of MAP programs in Sydney	n = 14	Alcohol dependent homeless individuals	N/A	N/A	Findings show that MAPs are significantly associated with reduced alcohol consumption	MAP programs were associated with better mental health stabilization	Findings show that MAPs are significantly associated with a reduction in emergency service contacts and hospital admissions	N/A
Muckle 2012 [68]	HIGH	To assess the effectiveness of managed alcohol regimens on their own or as compared to moderate drinking, screening and brief intervention using a harm reduction approach and traditional abstinence‐based interventions and no intervention	n = 0 (empty)	Vulnerable people aged 18 years or older who were at high risk for alcohol abuse, including those who are homeless, impoverished, mentally ill and past exposure to trauma	N/A	N/A	N/A	N/A	N/A	N/A
Nielsen 2018 [70]	CRITICALLY LOW	To review the current research evidence on harm reduction approaches and interventions for severe and chronic alcohol use in housing support initiatives.	n = 44	Individuals who are experiencing homelessness	In one trial, 8 men passed away while living in the MAP. Authors noted that clients were seriously ill prior to entering and their health deteriorated regardless of care. In another trial, 28 consecutive patients admitted to the program passed away in a two-year period. The most common diagnosis at admission were cirrhosis, malignancy, and HIV with an average time of 4 months from admission to death.	One trial found that medication compliance improved significantly among MAP clients with 88% taking their medication as prescribed 80% of the time. Two trials reported that many of the MAP clients saw improvements in their liver function tests since starting the program. One trial found that there was an increase in the number of MAP clients who met the criteria for alcohol-related liver damage.	Managed alcohol programs showed potential to increase alcohol consumption among clients. Two trials reported that clients who participated in MAP programs drank NBA alcohol on significantly less days and reported a significant reduction in withdrawal seizures than the control group. In one trial, clients reported a significant decrease in alcohol consumption, alcohol-related harms, as well as a significant improvement in mental health-related quality of life.	N/A	N/A	N/A
Pharmacological interventions for opioid use disorder										
Bahji 2018 [41]	CRITICALLY LOW	To identify pharmacological interventions for the prevention and treatment of opioid overdose	n = 8	Patients with an established opioid use disorder	In one trial, exposure to any opioid agonist treatment for more than 7 days significantly reduced the risk of mortality	N/A	Extended-release naltrexone was significantly associated with a higher percentage of opioid-negative urine drug test as well as a significant reduction in illicit opioid use. One trial found a positive effect of extended-release naltrexone on overdose events. There was a significant difference favouring the diacetylmorphine group on overdose events compared to the methadone or hydromorphone group Methadone was found to have greater positive effect on illicit drug use compared to forced withdrawal. However, there was no significant difference between groups on overdose events There was no significant difference between the buprenorphine and methadone groups on overdose events. There was no significant difference between the LAAM and methadone groups on overdose outcomes. However, the LAAM group reported significantly lower rates of illicit opioid use compared to the methadone group	N/A	N/A	Extended-release naltrexone was significantly associated with greater retention rates in treatment. Compared to hydromorphone or methadone, diacetylmorphine showed a significant improvement in retention in treatment as well as a significant reduction in illicit opioid use. Methadone was found to have greater positive effect on retention in treatment compared to forced withdrawal
Clark 2002 [42]	LOW	To compare the efficacy and acceptability of LAAM maintenance with methadone maintenance in the treatment of heroin dependence	n = 18	Heroin dependents or patients in opioid replacement therapy for heroin dependence	No significant between group differences were found on mortality of all causes	N/A	Continuous abstinence of at least 4 weeks was significantly lower among LAAM patients compared to methadone patients. No significant difference was found between groups on the cessation of all opioid substitution therapy.	N/A	N/A	Patients were more likely to have stopped using LAAM than methadone by the end of the study. Almost twice the number of LAAM patients dropped out due to side effects compared to methadone patients.
Ferri 2006 [43]	CRITICALLY LOW	To assess heroin prescription effectiveness	n = 4	Chronic heroin-dependent individuals	Two studies reported fatalities with no difference between groups and no relation to treatment	N/A	One study showed that heroin helped people avoid illicit opiates. One study did not find a significant difference. Two studies found no difference between groups on using other substances	N/A	N/A	Two studies found that heroin alone and heroin + methadone are better than methadone alone in retaining patients in treatment. In one study, patients in the heroin arm remained in treatment longer than the methadone arm.
Gowing 2011 [44]	HIGH	To assess the effect of oral substitution treatment on risk behaviours and rates of HIV	n = 38	Opioid dependent drug users	N/A	Six studies reported decreased HIV risk and positive HIV seroconversion after entry to substitution treatment	All studies showed that substitution treatments were beneficial in significantly decreasing the proportion of participants using illicit opioids, the frequency of injecting, and sharing injecting equipment.	N/A	N/A	N/A
Jones 2012 [45]	CRITICALLY LOW	To review literature regarding outcomes following maternal treatment with buprenorphine	n = 44 (Total)	Buprenorphine maintained pregnant women and their offspring exposed in utero to buprenorphine	N/A	N/A	No significant differences were found between buprenorphine and methadone on any drug use measures at time of delivery, whereas findings were inconsistent during pregnancy	N/A	N/A	No significant differences were found between buprenorphine and methadone on treatment completion.
Karki 2016 [46]	CRITICALLY LOW	To explore relevant literature regarding the possible impact on methadone maintenance treatment on HIV risk behaviours	n = 12	HIV high risk injection drug users	N/A	All studies reported a significant association between MMT and reduced sex and drug-related HIV risk behaviours	The majority of studies reported that MMT significantly reduced the likelihood of frequent heroin injection, syringe borrowing, non-fatal overdose and pubic injection	N/A	N/A	N/A
Kirchmayer 2002 [47]	CRITICALLY LOW	To evaluate the efficacy of naltrexone maintenance treatment in preventing relapse	n = 11	Heroin dependent in- and out-patients, or former heroin addicts dependent on methadone	N/A	N/A	In most cases, no significant difference between naltrexone versus placebo or other alternative treatment was found on the use of opioid under treatment	N/A	N/A	In most cases, no significant difference between naltrexone versus placebo or other alternative treatment was found on successful completion of treatment
Klimas 2019 [63]	LOW	To assess the efficacy of slow release oral morphine (SROM) as a treatment for opioid use disorder (OUD).	n = 4	Persons with opioid use disorder as defined in the DSM-IV	N/A	No difference in incidence of adverse events (81% SROM vs 79% methadone)	No difference between SROM and methadone in reducing opioid use (RR = 0.96; 95% CI: 0.61 to 1.52, p = 0.86, I^2^ = 50%)	In one study SROM was associated with fewer adverse mental symptoms	N/A	Difference in dropouts was not statistically significant between participants in the SROM vs methadone (RR = 0.98; 95% CI: 0.94 to 1.02, p = 0.34)
Larney 2014 [48]	MODERATE	To assess the efficacy and adverse events of naltrexone implants when used to treat opioid dependence	n = 9	Opioid dependents	No significant differences in mortality rates between naltrexone implants and TAU or buprenorphine maintenance treatment. Another trial reported no evidence of increased risk of death due to overdose after naltrexone treatment	One trial reported no opioid-related overdose requiring ED for patients with naltrexone implant or oral naltrexone. Another trial reported no significant differences in number of self-reported overdoses	Naltrexone implants significantly decreased opioid use compared to placebo or oral naltrexone. However, patients with naltrexone implants reported significantly higher use of non-opioid drugs than those on oral naltrexone	N/A	N/A	Two studies showed that naltrexone implants significantly increased treatment retention than placebo implants and oral naltrexone.
Lobmaier 2008 [49]	LOW	To evaluate the effectiveness of sustained-release naltrexone and its adverse effects	n = 17	Adults or adolescents with opioid dependence	N/A	N/A	No significant difference between groups was found on “wanting heroin” but a significant reduction favouring both naltrexone groups compared to placebo was found on “needing heroin”. No significant between group difference was found on severity of opioid and cocaine use	No significant between group difference was found on depression	N/A	No statistically significant difference in retention in treatment was found between those receiving naltrexone depot (lower or higher dose) or those receiving placebo. However, a significant difference between the high dose and the placebo groups was found on time to drop out
Maglione 2018 [64]	LOW	To evaluate the effects of MAT (using buprenorphine, buprenorphine plus naloxone, methadone, or naltrexone) for OUD on functional outcomes compared to wait-list, placebo, treatment without medication, any other comparator, or each other (e.g., buprenorphine vs naltrexone)	n = 40	Adults 18 years or older using medication-assisted treatment (MAT) for OUD—methadone, buprenorphine, buprenorphine plus naloxone, or naltrexone	N/A	Buprenorphine patients had a significantly lower prevalence of fatigue compared to methadone. No difference in insomnia between buprenorphine and methadone participants	N/A	N/A	N/A	N/A
Mattick 2009 [51]	LOW	To evaluate the effects of methadone maintenance treatment (MMT) compared with treatments that did not involve opioid replacement therapy	n = 11	Individuals with opioid dependence	No significant between-group difference was detected on mortality	N/A	Methadone was shown to significantly reduce heroin use compared to control conditions	N/A	N/A	Methadone had a superior retention rate compared with control conditions
Mattick 2014 [50]	LOW	To evaluate buprenorphine maintenance compared to placebo and to methadone	n = 31	Individuals dependent on heroin or other opioids	Three studies reported no deaths. One study reported a 20% mortality rate for the control group. One study reported two death with no relation to treatment condition.	N/A	Patients receiving high dosage of buprenorphine showed significantly less heroin use than the placebo group. Patients receiving medium dosage of buprenorphine showed significantly less cocaine and benzodiazepines use than the placebo group. No other significant differences were detected for different dosages.	N/A	N/A	Patients receiving flexible or low-dose methadone reported higher rates of retention in treatment than those with buprenorphine, whereas no significant differences were found on medium or high-dose groups. There was a significant benefit favouring patients receiving buprenorphine at any dose compared to those receiving placebo on treatment retention
Minozzi 2011 [52]	LOW	To evaluate the effects of naltrexone maintenance treatment versus placebo or other treatments in preventing relapse	n = 13	In-patients and out-patients dependent on heroin, or former heroin addicts dependent on methadone and participating in naltrexone treatment for opioid dependence	N/A	N/A	No significant difference between naltrexone versus placebo or no pharmacological treatment was found on abstinence. No significant difference between naltrexone versus psychotherapy was found on abstinence	N/A	N/A	No significant difference between naltrexone or naltrexone plus psychotherapy versus any control was found on retention in treatment
Platt 2017 [53]	MODERATE	To assess the effects of needle syringe programs and opioid substitution therapy, alone or in combination, for preventing acquisition of HCV	n = 28	People who inject drugs (opioids and/or stimulants)	N/A	Opioid substitution therapy (OST) was significantly associated with a reduction in HCV infection compared to no OST. High-coverage of needle syringe programs NSP was marginally associated with a reduction in HCV infection compared with lower coverage or no coverage, whereas no significant difference was found between low-coverage NSP versus no NSP. The combination of OST and NSP was significantly associated with a reduction in HCV infection.	N/A	N/A	N/A	N/A
Roozen 2006 [54]	CRITICALLY LOW	To summarize available and recent evidence on the effectiveness of naltrexone treatment	n = 7	Participants with alcohol or opiate abuse or dependence	N/A	N/A	Two studies reported no statistically significant medium- term difference on abstinence between naltrexone and the placebo groups, whereas one study reported a significant benefit favouring naltrexone. One study reported a significant benefit favouring naltrexone group compared to the placebo group on abstinence, whereas one study showed no significant difference No significant difference was found on continuous abstinence outcomes. There is a significant difference favouring the naltrexone group on drinking days compared to placebo. Abstinence was significantly higher for patients receiving naltrexone and supportive therapy.	N/A	N/A	There is a significant medium term benefit favouring patients receiving naltrexone on relapse rates compared to the placebo group There is moderate evidence favouring patients receiving naltrexone long term on relapse rates
Saulle 2017 [55]	LOW	To compare the effectiveness of opioid substitution therapy OST with supervised dosing relative to dispensing of medication for off-site consumption	n = 6	People “diagnosed as opioid dependent and receiving opioid substitution treatment with either buprenorphine or methadone	One study found that all-cause mortality was lower in the supervised methadone group. However, after adjustment insufficient evidence existed to support a protective effect.	Two trials found no significant difference between supervised and unsupervised therapy groups in serious adverse events requiring hospitalization	One study found no significant difference between supervised and unsupervised therapy in self-reported heroin use at three months or ASI’s composite score	N/A	N/A	There was no significant difference between between supervised and unsupervised therapy groups in retention rates across time
Simoens 2005 [56]	LOW	To evaluate the effectiveness of maintenance treatment with methadone or buprenorphine in treating opiate dependence	n = 48	Opiate dependent subjects aged 18 years old or over	N/A	N/A	Maintenance interventions with methadone or buprenorphine has been proven to be effective in reducing illicit opiate use and stimulating abstinence. No significant differences between methadone and buprenorphine were found in reducing illicit opiate use, cocaine use, or severity of withdrawal symptoms	N/A	N/A	Maintenance interventions with methadone or buprenorphine has been proven to be effective in promoting retention in treatment. Four studies found no significant difference between methadone and buprenorphine in retention in treatment, whereas three found additional benefit favouring methadone.
Sordo 2017 [57]	CRITICALLY LOW	To compare the risk for all cause and overdose mortality in people with opioid dependence during and after substitution treatment with methadone or buprenorphine and to characterise trends in risk of mortality after initiation and cessation of treatment.	n = 20	People with opiate dependence during and after substitution treatment with methadone or buprenorphine	Time spent in OST with methadone was associated with a significant reduction of mortalities. The mortality rate in treatment was less than a third of the rate out of treatment. Three studies showed that OST with buprenorphine could be associated with a reduction in mortality rates.	N/A	N/A	N/A	N/A	N/A
Standiford Helm 2008 [58]	CRITICALLY LOW	To evaluate and update the available evidence regarding the use of agonist/ antagonists to provide office- based opioid treatment for addiction.	n = 20	Patients with opioid dependence (whether they are in or out of treatment)	N/A	N/A	The combination of buprenorphine and naltrexone was found to significantly improve opioid dependence outcomes and had a significantly greater benefit on these outcomes compared to clonidine. Office-based treatment, levomethadyl, buprenorphine (with or without psychosocial treatments), and high dose methadone were found to be more effective than low dose methadone. No significant difference was found between methadone and buprenorphine. Tramadol compared favourably to buprenorphine in managing acute withdrawal symptoms/	N/A	N/A	As compared with low dose methadone, participants taking levomethadyl acetate had a higher rate of continuous abstinence from opioids, and those taking buprenorphine and high dose methadone had a trend towards higher rate of continuous abstinence.
Strang 2015 [59]	CRITICALLY LOW	To synthesise published findings for treatment with SIH for refractory heroin-dependence through systematic review and meta-analysis, and to examine the political and scientific response to these findings.	n = 6	Individuals with heroin dependence unresponsive to standard treatments	There was a positive but not significant effect favouring supervised injectable heroin SIH compared to oral methadone maintenance treatment on mortality	Five trials showed a significant higher risk of side effects in the SIH group compared to the oral MMT group	There was an overall positive effect favouring supervised injectable heroin SIH compared to oral MMT on illicit heroin use	N/A	N/A	Pooled analysis showed significant difference favouring SIH compared to oral methadone maintenance treatment on treatment retention
Thomas 2014 [60]	CRITICALLY LOW	To describe buprenorphine maintenance therapy and review available research on its efficacy	n = 19	Individuals with opioid dependence	N/A	N/A	The majority of studies reported a significant positive impact of buprenorphine maintenance treatment on illicit opioid use compared to placebo, lower dosage BMT or methadone maintenance treatment. However, when dosing the medication adequately, both buprenorphine and methadone showed comparable reduction in illicit opioid use.	N/A	N/A	Results indicate better treatment retention associated with methadone maintenance treatment. Rates of neonatal abstinence syndrome were similar for mothers treated with either buprenorphine or methadone. However, infant symptoms were less among the buprenorphine group
Weinmann 2004 [61]	CRITICALLY LOW	To assess the national and international literature on the effectiveness of substitution-based treatment	n = 13	Opiate addicts	N/A	N/A	Substitution therapy with methadone was found to be an effective strategy to reduce illicit drug use and improve the rehabilitation of opiate addicts	N/A	N/A	Speedy inclusion in the substitution therapy improved success of treatment
Wilder 2015 [62]	CRITICALLY LOW	To evaluate the discontinuation rates of MAT during pregnancy and in the immediate postpartum period. To examine what interventions (methadone or buprenorphine) improve treatment retention compared to standard care	n = 15	Pregnant and postpartum women with opioid use disorder	N/A	N/A	N/A	N/A	N/A	There is a scarce information on the range of prenatal and postnatal discontinuation rates, which limits generalizable findings. Duration of methadone treatment prior to delivery was inversely associated with risk for postpartum discontinuation of treatment
Pharmacologic agents for reversal of opioid poisoning										
Bahji 2018 [41]	CRITICALLY LOW	To identify pharmacological interventions for the prevention and treatment of opioid overdose	n = 4	Patients with an established opioid use disorder	N/A	N/A	There was no significant difference in efficacy between naloxone and physostigmine for in-hospital treatment of heroin overdose. There was no significant difference in efficacy between intranasal and intramuscular naloxone. There was no significant difference in efficacy between naloxone and nalmefene (1 or 2 mg).	N/A	N/A	N/A

### Pharmacological interventions for opioid use disorder

Reviews on pharmacological interventions reported on the use of methadone, buprenorphine, diacetylmorphine (heroin), levo-α-acetylmethadol (LAAM), slow release oral morphine and hydromorphone for treatment of opioid use disorder. Certainty of evidence from these reviews ranged from very low to moderate (see Table 4). One review [57], including one observational study among homeless populations, reported pooled all-cause mortality rates of 36.1 and 11.3 per 1000 person-years for participants out of and in methadone maintenance therapy respectively (rate ratio 3.20, 95%CI 2.65 to 3.86), and mortality rates of 9.5 per 1000 person years for those not receiving buprenorphine maintenance therapy compared to 4.3 per 1000 person-years among those receiving the therapy (rate ratio 2.20, 95%CI 1.34 to 3.61; GRADE certainty of evidence: very low). Higher rates of mortality were reported among heroin-dependent persons using LAAM (relative risk 2.28 (95%CI 0.59–8.9; GRADE certainty of evidence: low)) [42] and people chronically injecting opioids receiving diacetylmorphine (11 vs 3, p<0.05, no further data reported) [41] compared to methadone, however there were not enough deaths in these samples to draw any meaningful conclusions.

**Table 4:** GRADE evidence profiles for pharmacological interventions for opioid use disorder.

Certainty assessment							Summary of Findings				Certainty	Importance
№ of studies	Study design	Risk of bias	Inconsistency	Indirectness	Imprecision	Other considerations						
**Outcome: Mortality**												
All-cause mortality in and out of MMT (Source systematic review: Sordo, 2017, 16 cohort studies)												
16	Observational studies	Serious ^a^	Serious ^b^	Not serious	Not serious	None	Pooled all-cause mortality rates were 11.3 and 36.1 per 1000 person years in and out of methadone treatment (unadjusted out-to-in rate ratio 3.20, 95% confidence interval 2.65 to 3.86)				⨁◯◯◯ VERY LOW	CRITICAL
Overdose mortality in and out of MMT (Source systematic review: Sordo, 2017, 11 cohort studies)												
11	Observational studies	Serious ^a^	Serious ^c^	Not serious	Not serious	None	Pooled overdose mortality rates were 2.6 and 12.7 per 1000 person years in and out of methadone treatment (unadjusted out-to-in rate ratio 4.80, 2.90 to 7.96)				⨁◯◯◯ VERY LOW	CRITICAL
All-cause mortality in and out of BMT (Source systematic review: Sordo, 2017, 3 cohort studies)												
3	Observational studies	Serious ^a^	Not serious	Not serious	Not serious	Publication bias ^d^	Pooled all-cause mortality rates were 4.3 and 9.5 in and out of buprenorphine treatment (unadjusted out-to-in rate ratio 2.20, 1.34 to 3.61)				⨁◯◯◯ VERY LOW	CRITICAL
Overdose mortality in and out of BMT (Source systematic review: Sordo, 2017, 1 cohort study)												
1	Observational studies	Not serious	Not serious	Not serious	Not serious	None	In the single buprenorphine cohort there were 1.4 and 4.6 fatal overdoses per 1000 person years in and out of treatment				⨁ ⨁◯◯ LOW	CRITICAL
All-cause mortality in LAAM maintenance vs. methadone maintenance for heroin dependence (Source systematic review: Clark et al. 2002, 11 trials)												
11	Randomised trials	Serious ^i^	Not serious	Not serious	Serious ^j^	None	**Number of patients**		**Relative**	**Absolute**	⨁ ⨁◯◯ LOW	CRITICAL
							**LAAM**	**methadone**				
							**5/760**	**1/755**	**2.28 [0.59–8.90]**	**2 more per 1,000 (from 1 fewer to 10 more)**		
Mortality in methadone maintenance treatment vs. no methadone maintenance treatment												
4	Randomised trials	Not serious	Not serious	Not serious	Serious ^j^	None	**Number of patients**		**Relative**	**Absolute**	⨁ ⨁ ⨁◯ MODERATE	CRITICAL
							**methadone**	**No methadone**				
							**3/287**	**8/289**	**0.48 [0.10–2.39]**	**14 fewer per 1,000 (from 25 fewer to 38 more)**		
**Outcome: Morbidity**												
Hepatitis C (HCV) acquisition (Source systematic review: Platt, 2017, 12 cohort studies)												
12	Observational studies	Serious ^e^	Not serious	Not serious	Not serious	Large magnitude of effect ^f^				OST reduces the risk of HCV acquisition by 50% (risk ratio (RR) 0.50, 95% confidence interval (CI) 0.40 to 0.63, I2 = 0%, 12 studies across all regions, N = 6361),	⨁ ⨁◯◯ LOW	CRITICAL
Psychological Morbidity (Source systematic review: Standiford Helm 2008, 1 RCT Mattick 2003)												
1	Randomised trials	Not serious	Not serious	Not serious	Serious ^g^	None	There were significant overall improvements, but no difference between groups, in psychological morbidity (GHQ and SCL90R) [No further data reported].				⨁◯◯◯ VERY LOW	CRITICAL
**Outcome: Substance use**												
Reduction in non therapeutic opioid use (Source systematic review: Mattick 2014, 4 RCTs)												
4	Randomised trials	Not serious	Serious ^h^	Not serious	Not serious	None	High-dose buprenorphine (≥ 16 mg) was more effective than placebo in suppressing illicit opioid use measured by urinalysis in the trials (3 studies, 729 participants, SMD -1.17; 95% CI -1.85 to -0.49) Notably, low-dose, (2 studies, 487 participants, SMD 0.10; 95% CI -0.80 to 1.01), and medium-dose, (2 studies, 463 participants, SMD -0.08; 95% CI -0.78 to 0.62) buprenorphine did not suppress illicit opioid use measured by urinalysis better than placebo.				⨁ ⨁ ⨁◯ MODERATE	CRITICAL

Explanations

a. Several studies of low quality resulting from confounding bias and differential loss to follow up.

b. All-cause mortality rates varied widely across the 16 methadone cohorts (overall I2 = 98%, P<0.001), although rates were consistently higher out of treatment than in treatment

c. There was moderate heterogeneity between studies in mortality rates in treatment (I2 = 66%, P = 0.001) and strong heterogeneity in rates out of treatment (I2 = 97%, P<0.001), with significantly higher rates out of treatment among methadone patients in specialist services than in primary care

d. There was some evidence of small study effects on all-cause mortality (P = 0.05), with higher rates in small cohorts that mostly enrolled opioid injectors who were positive for HIV

e. Downgraded one level due to overall moderate risk of bias in 2 studies, overall serious risk of bias in 6 studies, 2 studies at overall critical risk of bias in 2 studies; not enough information to make judgment in 2 studies.

f. Upgraded one level due to large magnitude of the effect: RR: 0.5.

g. Imprecision downgraded due to lack of data reported. No measures reported, no effect estimates available

h. Inconsistent results related to high-, medium-, and low-dose of buprenorphine

i. The method of randomization and allocation concealment was not stated in the majority of studies, possibly due to the era in which these studies were published. It is it not known whether blinding was effective since no studies provided data to support the effectiveness of the blind

j. Too few events.

**Fig 2:**
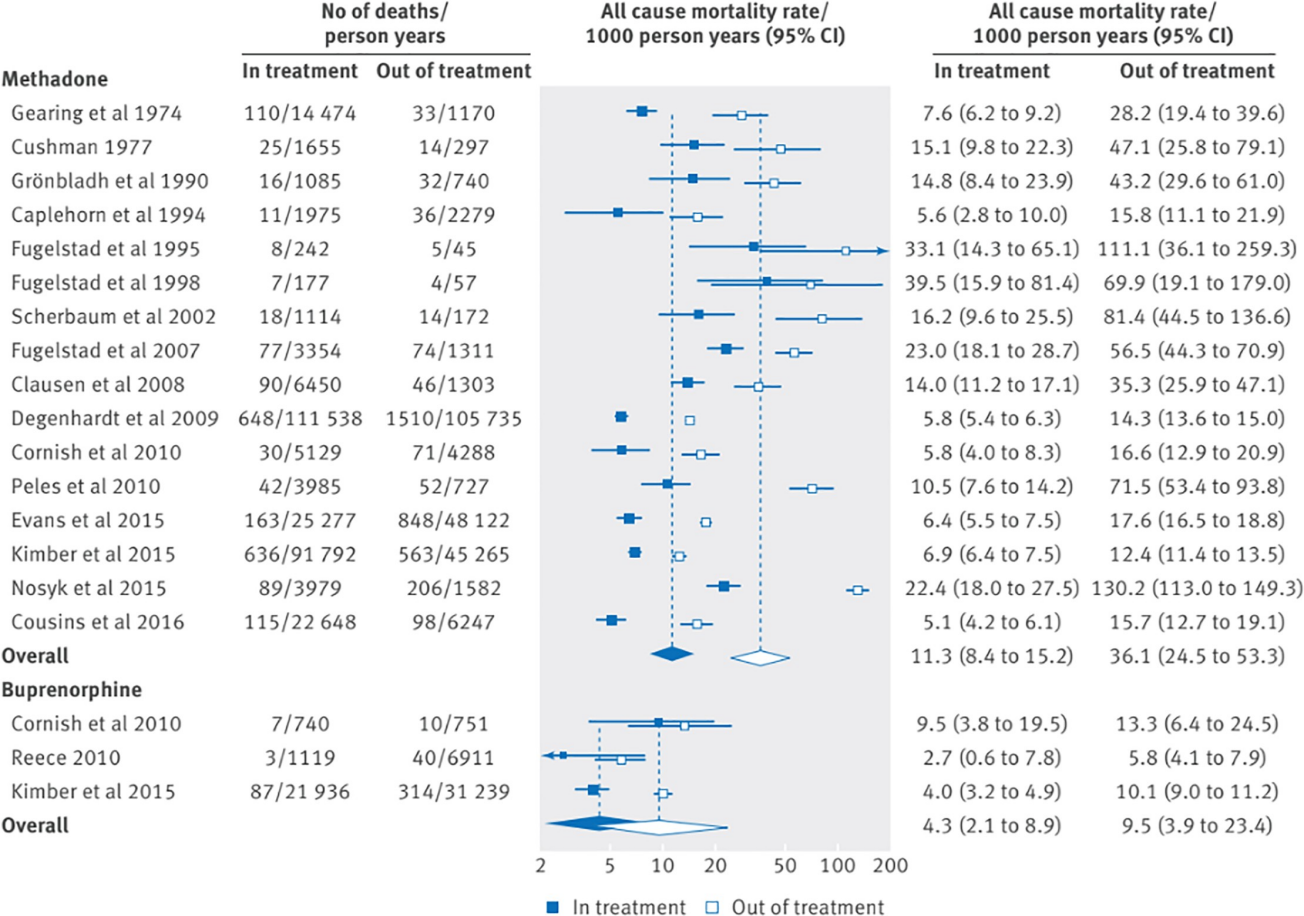
All-cause mortality rates in and out of methadone and buprenorphine treatment (Sordo et al., 2017; creative commons attribution non commercial (CC BY-NC 4.0) license).

**Fig 3:**
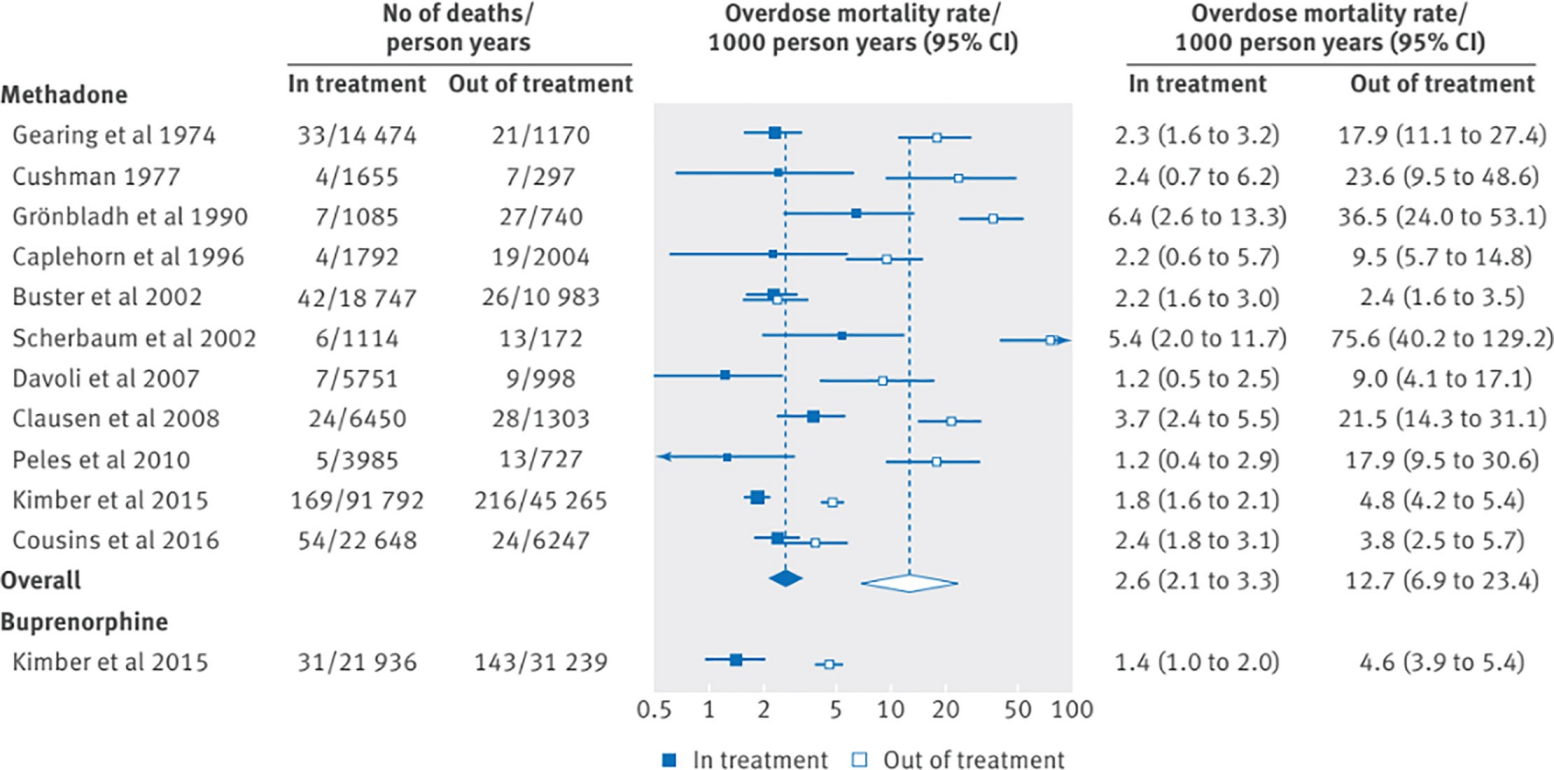
Overdose mortality rates in and out of methadone and buprenorphine treatment (Sordo et al., 2017; creative commons attribution non commercial (CC BY-NC 4.0) license).

Overdose-specific mortality rates were similarly affected, with pooled overdose mortality rates of 12.7 and 2.6 per 1000 person years for participants out of and in methadone maintenance therapy (GRADE certainty of evidence: very low), and rates of 4.6 and 1.4 per 1000 person years out of and in buprenorphine maintenance therapy (GRADE certainty of evidence: low) [57] (See Figs 2 and 3). Conversely, in an older review among heroin-dependent persons including fewer studies, compared to non-pharmacological approaches, methadone maintenance therapy had no statistically significant effect on mortality (relative risk = 0.48; 95%CI: 0.10–2.39; GRADE certainty of evidence: moderate) [51].

With respect to morbidity, pharmacological interventions for opioid use disorder reduced the risk of Hepatitis C (HCV) acquisition (risk ratio 0.50, 95%CI 0.40–0.63; GRADE certainty of evidence: low) [53] and HIV infection [44]. Because of the high risk of bias and variability in several aspects of studies examining the risk of HIV infection, combined totals were not calculated [44]. Methadone was associated with more adverse events compared to buprenorphine, including sedation [50,60], but not compared to slow release oral morphine (81% SROM vs 79% methadone, p = 0.62 [no further data reported])[63]. Buprenorphine patients had a significantly lower prevalence of fatigue (15.5% vs 25.1%; risk ratio 0.62; 95% CI 0.41–0.95; GRADE certainty of evidence [as reported in [64]: moderate) but no difference in insomnia (risk ratio1.12 95%CI 0.78–1.62) compared to methadone patients [64].

Therapies for opioid use disorder among general populations were associated with statistically significant reductions in non-prescribed opioid use [41,44,45,50,51,58,60], injection drug use and sharing of injection equipment [44,46]. Studies conducted among homeless populations demonstrated an association between prescribed use of diacetylmorphine or methadone and reductions in non-prescribed drug use or more general illegal activity [71]. Several studies also reported reduced risk behaviours such as having multiple sexual partners, unprotected sex or participation in drug-related crime [46]. Buprenorphine and methadone further showed improvements in social functioning, physical health, and psychological morbidity [58].

Findings on access to care and retention in treatment were mixed. A review on buprenorphine treatment found that it was associated with expanded access to treatment for patients who may not enroll in methadone clinics, and facilitated earlier access to treatment for patients who have more recently initiated opioid use [58]; however there did not appear to be any effect on retention whether buprenorphine was dosed under supervised or unsupervised settings (risk ratio 0.99, 95% CI 0.88–1.12) [55]. A systematic review highlighted that methadone maintenance therapy was more effective than non-pharmacological approaches in retaining heroin-dependent patients in treatment (risk ratio 4.44, 95% CI:3.26–6.04) [51]. Of note, methadone maintenance therapy use was negatively associated with homelessness in a separate observational study (odds ratio 0.29, 95% CI 0.17–0.49) [72]. One review reported that difference in dropouts was not statistically significant between participants in slow release morphine vs methadone (risk ratio 0.98; 95% CI: 0.94–1.02, p = 0.34) [63]. The relative superiority of one pharmacological agent over another on retention outcomes remains unclear.

Finally, three reviews reported on naltrexone treatment. No findings were specific to homeless populations. Naltrexone was associated with few opioid overdose events [41,48]. Naltrexone implants were significantly better than placebo (risk ratio 0.57; 95% CI 0.48–0.68) or oral naltrexone (risk ratio 0.57; 95% CI 0.47–0.70) at reducing opioid use [48]. Extended-release naltrexone led to significantly greater retention in treatment, significantly greater proportions of opioid-negative urine drug tests, and significantly greater reductions in illicit opioid use compared to treatment as usual [41]. Results of a meta-analysis were not statistically significant for the successful completion of treatment (odds ratio 0.78; 95% CI 0.34–1.75) when comparing oral naltrexone versus placebo [47]. Naltrexone implants showed significantly better treatment retention than placebo implants (risk ratio 3.20; 95% CI 2.17–4.72) and oral naltrexone (risk ratio 3.38; 95% CI 2.08–5.49) [48].

### Pharmacologic agents for reversal of opioid overdose

We identified one review reporting the effectiveness of naloxone among populations experiencing opioid use disorder, [41] which identified four trials that investigated naloxone in the treatment of suspected or established opioid overdose. Three trials involved prehospital treatment of suspected opioid overdose [73–75]. One trial [76] compared intravenous naloxone to physostigmine for in-hospital treatment of heroin overdose, finding no significant difference in efficacy. Two trials [74,75] compared intranasal to intramuscular naloxone, finding no significant difference in efficacy. However, Kerr et al. [75] reported that supplementary naloxone was administered to fewer patients who received intramuscular naloxone (IN 18.1%; IM 4.5%; difference: 13.6%, 95% CI 4.2–22.9). Neither of these trials reported on housing stability of the patients, however, Kelly et al. [74] reported that more patients were treated in public places such as a park or street than in private residences. One trial [73] compared intravenous naloxone to nalmefene (1 or 2mg), finding no significant difference in efficacy. Adverse events occurred in 30.9% (2mg nalmefene), 15.9% (1 mg nalmefene), and 15.5% (naloxone) of patients; group differences were not statistically significant (p>0.08; [no further data reported]). No reviews on naloxone reported on substance use, mental health, or access to care outcomes.

### Supervised consumption facilities

Our search identified three reviews providing very low to low certainty evidence (see Table 5) that examined supervised consumption facilities [65–67]. Vancouver’s INSITE, the first legal supervised drug injection site in North America, and the Sydney Medically Supervised Injection Centre (MSIC) are two SCFs which have been extensively evaluated in these reviews. Vancouver’s INSITE was established in 2003, and it is estimated that 20% of its clients are absolutely homeless, with many more living in single-residence rooms [77]. In Sydney, the MSIC facility has been operating since 2001 with an estimated 11% of its clients in unstable accommodations [78]. In both sites, no deaths from opioid overdose were reported [66]. As well, there was a significant decrease in the number of deaths in the immediate vicinity of the Sydney SCF, from an average of 4 to 1 deaths per month (p<0.001) [65].

**Table 5:** GRADE evidence profiles for SCFs.

Certainty assessment							Summary of Findings	Certainty	Importance
№ of studies	Study design	Risk of bias	Inconsistency	Indirectness	Imprecision	Other considerations			
**Outcome: Mortality**									
Lethal overdoses in SCF proximity (Source systematic review: Kennedy 2017; 1 observational trial)									
1	Observational studies	Not serious	Not serious	Not serious	Not serious	None	35% decrease in the number of lethal overdoses within 500 metres of the SCF from 253.8 (187.3 to 320.3) to 165.1 (108.8 to 221.4) deaths per 100,000 person-years (RD 88.7 (1.6 to 175.8) per 100 000 person years; p = 0.048), compared to the rest of the city: 9.3%, from 7·6 (6.2 to 9.0) to 6·9 (5.5 to 8.4) deaths per 100 000 person-years (p = 0·490).	⨁⨁◯◯ LOW	CRITICAL
Lethal overdoses avoided each year (Source systematic review: Kennedy 2017; 2 observational trials)									
2	Observational studies	Not serious	Not serious	Serious ^a^	Not serious	None	In Vancouver, the SCF avoided 1004 overdoses over 4 years, including 453 life-threatening overdoses, suggesting that between 2 and 12 cases of lethal overdose might have been avoided each year. A study from Germany estimate that at least 10 deaths are prevented by SCF each year.	⨁◯◯◯ VERY LOW	CRITICAL
Deaths during study period (Source systematic review: Kennedy 2017; 2 observational trials)									
2	Observational studies	Not serious	Not serious	Not serious	Not serious	None	There were 336 reported overdoses within the SCF over a four year period, and no deaths. In another study, over a 17 month period, there were 409 overdoses and no deaths.	⨁ ⨁◯◯ LOW	CRITICAL
**Outcome: Morbidity**									
HIV cases averted (Source systematic review: Kennedy 2017; 2 observational trials)									
2	Observational studies	Not serious	Serious	Serious ^a^	Not serious	None	A mathematical simulation estimates that 1191 incident HIV cases were averted over 10 years. Another mathematical model estimated that the SCF prevents 22 incident HIV infections per year.	⨁◯◯◯ VERY LOW	CRITICAL
HCV cases averted (Source systematic review: Kennedy 2017; 1 observational trial)									
1	Observational studies	Not serious	Not serious	Serious ^a^	Not serious	None	A mathematical simulation estimates that 54 incident HCV cases were averted over 10 years	⨁◯◯◯ VERY LOW	CRITICAL
**Outcome: Substance use**									
Injection drug use initiation (Source systematic review: Kennedy 2017; 1 observational trial)										
1	Observational studies	Not serious	Not serious	Not serious	Not serious	None	Among the entire population of SCF users in Vancouver (n = ~ 5000), the estimated number who may have initiated injection drug use inside the SCF since the SIF opened was 5 (95% CI 2–12), which is comparatively lower than the expected rate of initiation into injection drug use among local street-involved youth during a similar follow-up period (100 initiations; 95% CI 81–122)	⨁ ⨁◯◯ LOW	CRITICAL

Explanations

a. Mathematical simulations providing indirect evidence

The establishment of INSITE in Vancouver led to a 35% decrease in the number of fatal opioid overdoses within 500 meters of the SCF (from 253.8 to 165.1 deaths per 100,000 person-years; p = 0.048), compared to 9% in the rest of the city [65]. There were 336 reported opioid overdose reversals in 90 different individuals within the Vancouver SCF over a four-year (2004–2008) period [66]. Simulation studies estimate that, over the same four-year period, up to 1004 opioid overdoses may have been avoided in Vancouver, including 453 life-threatening opioid overdoses [66], suggesting that between 2 and 12 cases of lethal opioid overdoses might have been avoided each year (GRADE certainty of evidence: very low) [65]. Similarly, there were 409 opioid overdose reversals associated with the establishment of MSIC in Sydney over 17 months, and similar protective effects of SCFs have been reported in Germany [65]. There were no substantial changes in rates of relapse into injection drug use, ceasing injection, ceasing binge drug use, or participation in methadone maintenance therapy after the Vancouver SCF opened [65]. The rate of recent initiation into injection drug use among SCF clients was markedly lower than the estimated background community-level rate of injection initiation [65]. Frequent SCF use in Sydney was positively associated with experiencing a non-fatal opioid overdose within the SCF (AOR = 6.1; 95% CI 4.3–8.6) [65]. There was also a significant decrease in opioid overdose emergency department presentations, from an average of 11 to 7 per month (35% reduction, p<0.001) [65].

Observational studies conducted in Vancouver and Sydney showed that regular use of SCFs was associated with decreased syringe sharing (aOR = 0.30, 95%CI = 0.11–0.82), syringe reuse (aOR = 2.04, 95%CI = 1.38–3.01), and public-space injection (aOR = 2.79, 95%CI = 1.93–3.87) [66]. The SCF in Vancouver was associated with reductions in the number of people injecting in public [65]. It has been noted that factors associated with public injection in Vancouver include homelessness (aOR = 3.1, 95%CI = 1.46–6.58) [66]. One review suggested that SCFs prevented incident HIV and HCV infections (GRADE certainty of evidence: very low) [65], but the exact number of cases averted depends on varying mathematical model assumptions.

Reviews noted that SCFs may have improved access to care for vulnerable populations; their advantages included competent staff and non-judgmental transfer to a hospital if necessary, education on safer injection, and transfer to other medical and social structures [66]. SCFs mediated access to ancillary services (e.g. food and shelter) and fostered access to broader health supports [67]. SCF attendance was associated with an increase in referrals to an addiction treatment center and initiation of methadone maintenance therapy (aHR = 1.57, 95%CI = 1.02–2.40) [66]. A study in Sydney found that frequent SCF use was positively associated with referral to addiction treatment, but not addiction treatment uptake [65]. In a Danish study where 40% of participants were unstably housed, being advised to seek treatment for a medical condition by SCF staff was associated with an increased likelihood of receiving treatment (51.3 vs. 25.7%, p = 0.003 [no further data reported]) [65]. On average, 21% of individuals attending the SCF in Vancouver wanted, but were unable to access, substance use treatment. The main obstacle in access was the waiting list, and homelessness was significantly associated with lack of access (OR = 1.47, 95%CI = 1.09–1.98) [66]. No systematic reviews reported on the effects of SCFs on mental health outcomes.

### Managed alcohol programs

We identified one Cochrane systematic review [68] and two grey literature systematic reviews [69,70] examining the effects of managed alcohol programs (MAPs) for homeless populations. There was a lack of high-quality evidence in the peer reviewed literature for this intervention [68]. Existing evidence consisted largely of uncontrolled evaluations of small scale pilot programs. Few studies reported on deaths among MAP clients [70]. In a case study of a Canadian MAP, “out of few dozen men who cycled through the program, eight clients of the MAP had passed away since its opening in 2006” [70]. Additionally, a MAP within a shelter-based palliative care program had 28 consecutive patients die between 2001 and 2003 [70]. Impacts of MAPs on hepatic function are mixed, with some studies reporting improvement in hepatic laboratory markers over time, and others showing increases in alcohol-related hepatic damage [70], however this may have occurred regardless of entry into a MAP. This evidence suggested that MAPs result in stabilized alcohol consumption and can facilitate engagement with medical and social services [69]. MAP clients also reduced their consumption of non-beverage alcohol products, liquids containing a form of comparatively harmful forms of alcohol that were not intended for human consumption (e.g., mouthwash, hand sanitizer, etc.) and were consumed instead of beverage alcohol for the purposes of intoxication [70]. MAP clients experienced significantly fewer social, health, safety, and legal harms related to alcohol consumption [70]. Individuals participating in MAPs demonstrated fewer hospital admissions and a 93% reduction in emergency service contacts [69]. MAPs also promoted improved or stabilized mental health [69] and medication adherence [70]. No systematic reviews reported on retention in treatment outcomes.

## Discussion

We conducted a systematic review of systematic reviews to understand the effectiveness of substance use interventions of relevance to homeless and vulnerably housed populations with concomitant substance use disorder. To our knowledge, this is the first systematic review on harm reduction approaches and pharmacological interventions for this population. While our review did not focus exclusively on studies among populations experiencing homelessness, people experiencing homelessness and people who use substances both face considerable social marginalization, multiple physical and/or mental health concerns, and high rates of premature morbidity [79]. Our review found that these interventions reduce harms associated with substance use and mitigate morbidity and mortality [41,44,45,50,51,53,57,58,60,65,66]. Since homelessness and unstable housing are factors that continue to be associated with public injection, infection, and reduced access to care [9,80,81], this further highlights the importance of offering low threshold services in close proximity to homeless populations and integrating housing supportive services for individuals who are homeless and have concomitant substance use disorders [82].

Several studies on pharmacological interventions demonstrated improved outcomes for mortality, HCV and HIV acquisition, psychological morbidity and non-prescribed opioid use [44,53]. Results suggest that buprenorphine and methadone are the most effective pharmaceutical agents to address mortality and morbidity among people who use substances [50,57,60]. However, these options may not be feasible for many people experiencing homelessness as they may face additional barriers when accessing opioid agonist therapy (i.e. accessing pharmacy daily, attending regular appointments) compared to those who use substances and are stably housed [83–86]. Much of the included evidence in this review took place prior to the current opioid epidemic, thereby missing important contextual factors related to treatment need and acceptability for homeless populations. Emerging evidence suggests that injectable heroin and injectable hydromorphone are both acceptable and associated with improved outcomes for people who are treatment-refractory [87]. Furthermore, the prevalence of stigma among health care providers towards people who use substances is well-documented and can result in barriers to healthcare access and poor health outcomes [88]. Individual patient characteristics and preferences should be considered when choosing a first-line opioid agonist treatment, including risk-factors for treatment drop-out such as unstable housing, lack of social support, and concurrent mental illness [89]. Other low-threshold pharmaceutical options are being adopted, including slow release oral morphine, diacetylmorphine, and injectable hydromorphone, which warrant ongoing pragmatic study, particularly with homeless and vulnerably housed populations. Traditionally, opioid use disorder pharmacotherapy was prescribed by specialists, however as with other chronic conditions, opioid use disorders can be managed in a primary care setting where primary care teams and clinician support and training can facilitate improved outcomes [89,90].

Non-prescription use of opioids and the resulting increase in overdose-related deaths has become a global issue over the past decade. Recent literature continues to highlight the importance of naloxone in averting overdose-related deaths [91,92]. Though we included naloxone as a pharmacological agent in our review, we did not search specifically for take-home naloxone programs as an intervention, and there are existing reviews documenting the value of these programs [93,94]. There is yet to be a systematic review considering their use among homeless and vulnerably housed populations, despite their disproportionate representation in the overdose epidemic [95,96]. However, recent studies indicate that take-home naloxone education programs are feasible within existing settings offering services to homeless and vulnerably housed populations [97] and the wider community [98,99].

Homeless and vulnerably housed populations are at risk of public injection and other high-risk consumption behaviours [100]. Our findings highlight that SCFs had positive impacts on fatal overdose prevention, public injecting and other high-risk consumption, which constitute an important mechanism for drug use-associated morbidity and mortality [101]. Additionally, SCFs can act as a bridge to other health and social services for those who seek them, although clients are often faced with long wait-lists to obtain services and treatment [65]. While public perception persists that SCFs may increase non-prescribed substance use and local crime and discourage individuals from seeking addiction treatment, such concerns are not supported by existing evidence [65,66]. The effective implementation of SCFs is unfortunately influenced by mixed public perceptions [102–106]. Finally, most formal evaluations, including those captured by this review, are of stand-alone SCF sites. In light of the current overdose epidemic, current initiatives may include integrated service models, mobile sites and virtual sites [107,108]. Future research examining the experiences of homeless or vulnerably housed persons engaging with these sites, with considerations of their acceptability and barriers/facilitators in accessing them, is needed to guide best practice.

Though a number of publications document their feasibility and acceptability, we could not identify any systematic reviews of high-quality comparative evidence (e.g. randomized trials) on MAPs. However, a national Canadian evaluation of MAPs is currently underway [109]. Previously, studies examining MAPs were largely conducted as case studies and small sample pilot projects [68], however, these studies demonstrated that such programs facilitate stabilization by providing a safe space that decreases the risks associated with substance use, promotes social and cultural reconnections, and provides an environment that fosters self-change [110–112]. Recent systematic reviews from grey literature conclude that few studies report on deaths of MAP clients, however many clients of MAPs have advanced, life-limiting illness prior to entering the program, and their health may deteriorate despite the best efforts of the health care providers [70], therefore rendering studies on mortality difficult to conduct and interpret. Moreover, reviews are often unable to provide high quality evidence on MAPs because of ethical and logistical barriers which need to be overcome in studying such vulnerable heterogeneous populations with complex needs [69].

### Strengths and limitations

We conducted a structured systematic search, used high quality methods to synthesize systematic reviews, included results in meta-analyses where appropriate, and used GRADE methods to assess the certainty of the effects. One of the strengths of this review is its ability to capture a wide body of evidence regarding our selected interventions. Several limitations should also be acknowledged. Due to the lack of existing trials studying these interventions among homeless and vulnerably housed populations (indeed, randomized trials of harm reduction interventions have particular ethical concerns), we expanded our search to the general population. The GRADE certainty of the evidence for health system and public health interventions is often low or very low [113], and this phenomena is replicated in our review. While we extracted homelessness and housing stability patient characteristics when possible, population-specific factors must be considered when interpreting our findings. Additionally, this review only included reviews that followed a systematic methodology, and excluded primary studies. A weakness inherent to secondary analysis is that our review may be limited by the decisions and potential biases of the authors of the included studies on the reporting of results. This may have impacted the variety of studies found and excluded the most recent primary studies in the field. We also recognize that grey literature searches have limitations, including the possibility that existing evidence is missed. While many homeless individuals were included in certain primary studies of pharmacological programs, homeless-specific results may have been omitted from the original reviews as a result of selective reporting. In addition, many of our included reviews are of low quality, since we did not exclude any reviews on the basis of methodological quality. The low scoring quality of these reviews indicates that they have one or more critical flaws in their methodology, as defined by AMSTAR 2 [38], and should not be relied on to provide an accurate and comprehensive summary of the available studies. For the majority of the included reviews these critical weaknesses were due to a lack of explicit reference to a review protocol and/or omission of a list of excluded studies. Adherence to a well-developed protocol reduces the risk of bias in the review, while excluded studies should be accounted for fully by review authors, otherwise there is a risk that they remain invisible and the impact of their exclusion from the review is unknown [38]. This lack of transparency means that these systematic review findings should be considered with reservations. Therefore, future reviews should aim to explicitly mention and adhere to a protocol and report all studies under consideration to improve their overall reporting quality. Finally, it is important to note limitations in the generalizability of our findings from the range of social and geographical settings reviewed. For example, data from Canada, Australia, the USA and UK may reflect weather extremes, universal healthcare or availability of low-threshold services not relevant to other countries, and vice versa.

### Implications for future research

While this review gathers a robust body of evidence of the effects of harm reduction and pharmacologic interventions on the health and social outcomes of people who use drugs who are homeless or vulnerably housed, more high quality research is required on MAPs, the scaling up of SCFs in a variety of settings such as supportive housing environments or as part of healthcare centres, as well as the types and ideal prescribing strategies of pharmacological interventions. Longer follow-up periods are also required to determine which interventions maintain their improvements over time. In order to inform decisions on resource allocation by public health and governmental bodies, future studies should continue to assess the cost-effectiveness of these interventions by evaluating and comparing the cost of their implementation against their benefits at the individual and societal levels [114–118]. In addition, more research looking at the effects of these interventions on specific subgroups of homeless populations that were not captured by this review, such as youth, women and Indigenous populations. As the value of peer-support and lived experience in intervention design and implementation is increasingly recognized [119], their role should be evaluated in future harm reduction interventions. Furthermore, additional research among homeless populations is needed to contextualize our findings and address the unanswered questions regarding the prescribing strategies of pharmacological interventions and implementation of harm reduction programs for homeless and vulnerably housed populations.

## Conclusions

Homeless and vulnerably housed populations experience high rates of substance use. Our review found that harm reduction strategies and pharmacologic interventions for opioid use disorder limit or prevent infectious diseases, other chronic conditions and opioid overdose while improving retention and access to care. Policy and civil society debates on the role of harm reduction may constitute barriers to implementation. The disproportionate levels of harm experienced by the most at-risk and highly stigmatized populations, including people who are homeless, heralds a call to action. Policy makers need to address growing calls by a number of organizations and people who use drugs for the decriminalization of drugs, as this reduces the harm caused by prohibition and may encourage more people to access health services while reducing the burden on the criminal justice system, and improve morbidity, mortality and substance use outcomes [120]. Finally, allocating resources to structural conditions that homeless people who use substances face, such as housing, trauma and poverty, has the potential to further reduce harms associated with the risk environment [121–123]. Primary care practitioners may benefit from incorporating harm reduction interventions into their practice to reduce morbidity in homeless populations. However, stigma, lack of available services and not knowing what services are available to homeless populations are often barriers to providing care, and instill bias in the care that is given [124]. Concurrently integrating harm reduction programs in primary healthcare services, building the capacity of staff working in these settings, and changing the culture within organizations has the potential to lead to sustained improvements of health and social outcomes [125].

## Supporting information

S1 File PRISMA checklist. (PDF)

S2 File Search strategy. (PDF)

S3 File AMSTAR II assessments. (PDF)

S4 File Citation matrix. (PDF)

S5 File List of excluded studies. (PDF)
