# Supplementary material for: The effectiveness of substance use interventions for homeless and vulnerably housed persons: A systematic review of systematic reviews on supervised consumption facilities, managed alcohol programs, and pharmacological agents for opioid use disorder
Source: PLoS One. 2020 Jan 16;15(1):e0227298. doi: 10.1371/journal.pone.0227298 (PMC6964917; doi:10.1371/journal.pone.0227298)
Supplement: S2 File — (PDF) [file pone.0227298.s002.pdf]

Appendix II: Inclusion Criteria

**Inclusion Criteria**	**Definitions**	
Population	People experiencing homelessness and vulnerable housing.	
Interventions	Supervised consumption facilities	Legally sanctioned facilities where people who use substances can consume pre-obtained substances under supervision (Drug Policy Alliance, 2018). There exists various terminologies for these facilities, including: supervised injection facilities (SIF), supervised consumption sites (SCS), medically supervised injection centres (MCIS), among others.
	Managed alcohol programs	Shelter, medical assistance, social services and the provision of regulated alcohol to help residents cope with severe alcohol use disorder (Shepherds of Good Hope Foundation, n.d.)
	Pharmacological interventions for opioid use disorder	Opioid therapy medications including methadone, buprenorphine, diacetylmorphine, levo-α-acetylmethadol (LAAM) and naltrexone.
	Pharmacologic agents for reversal of opioid overdose	Opioid antagonist administered intravenously or intranasally, e.g. naloxone.
Comparison	No intervention, standard intervention, alternative intervention, treatment as usual.	
Outcomes	Mortality, morbidity, substance use, mental health, access to care, retention in treatment	
Study design	Systematic review. Exclude all other study designs.	
**Exclusion Criteria**	**Justifications**	
Reviews that focus on low- and middle-income countries	Due to the variability in access to resources and supports in comparison to that in a high-income country vary greatly. We feel that the settings are different and should be synthesized separately.	
Reviews that focus on Indigenous populations	The analysis of the interventions tailored to this population will be covered by an Indigenous research group.	
Reviews which focus on incarcerated populations	Not generalizable to the non-incarcerated homeless population.	
Reviews which exclusively report on	Abstinence-based approaches are outside of the scope of this review.	

interventions for detoxification	
