# Supplementary material for: The effectiveness of substance use interventions for homeless and vulnerably housed persons: A systematic review of systematic reviews on supervised consumption facilities, managed alcohol programs, and pharmacological agents for opioid use disorder
Source: PLoS One. 2020 Jan 16;15(1):e0227298. doi: 10.1371/journal.pone.0227298 (PMC6964917; doi:10.1371/journal.pone.0227298)
Supplement: S3 File — (PDF) [file pone.0227298.s003.pdf]

Appendix III: Search Strategy

Database: Embase Classic+Embase <1947 to 2018 July 05>, Ovid MEDLINE(R) ALL <1946 to July 05, 2018>, PsycINFO <1806 to July Week 1 2018>, Joanna Briggs Institute EBP Database - <Current to June 27, 2018>, EBM Reviews - Cochrane Database of Systematic Reviews <2005 to July 5, 2018>, EBM Reviews - Database of Abstracts of Reviews of Effects <1st Quarter 2016>

Search Strategy:

----

1 drug users/ (106193)

2 exp *substance-related disorders/ (336853)

3 exp *alcohol-related disorders/ (159374)

4 alcoholics/ (127046)

5 exp *opioid-related disorders/ (25902)

6 exp *substance withdrawal syndrome/ (28023)

7 ((Illicit or injection or intravenous or iv or parenteral) adj2 drug use$).ti,kf. (11304)

8 ((Illicit or injection or intravenous or iv or parenteral) adj2 drug use$).ab. (37987)

9 (pwud or pwid).tw,kf. (3324)

10 or/1-9 (518390)

11 substance abuse treatment centers/ (13062)

12 needle-exchange programs/ (28591)

13 harm reduction/ (10064)

14 (supervised adj2 (consumption or drug or injection or treatment)).tw,kf. (1789)

15 ((needle? or syringe?) adj2 exchange).tw,kf. (4166)

16 or/11-15 (54427)

17 alcohol drinking/pc (5135)

18 *alcoholism/rh, th (17940)

19 (managed adj2 (alcohol or drinking)).tw,kf. (99)

20 (supervised adj2 (alcohol or drinking)).tw,kf. (39)

21 (alcohol$ and (manag$ or reduc$ or treat$) and (abus$ or delirium or withdrawal)).tw,kf. (62932)

22 ((alcohol or ethanol) adj2 (adminstr$ or administer$ or intravenous or iv or i v or prophyl$ or prescri$ or protocol? or provid$ or provision)).tw,kf. (12883)

23 or/17-22 (94502)

24 exp *opioid-related disorders/dt, pc, rh, th (12196)

25 substance abuse, intravenous/dt, pc, rh, th (2856)

26 narcotics/rh, tu (3559)

27 hydromorphone/ad, tu (1081)

28 methadone/ad, tu (9046)

29 naloxone/ad, tu (4867)

30 naltrexone/ad, tu (3467)

31 (((buprenorphine or diacetylmorphine or heroin or hydromorphone or methadone or morphine or opiate? or opioid$) adj (maint$ or replace$ or substitut$)) and (dependen$ or management or therap$ or treatment$)).ti,kf. (5732)

32 (((buprenorphine or diacetylmorphine or heroin or hydromorphone or methadone or morphine or opiate? or opioid$) adj (maint$ or replace$ or substitut$)) and (dependen$ or management or therap$ or treatment$)).ab. (14648)

33 or/24-32 (40355)

34 exp program evaluation/ (105081)

35 ((effectiveness or improve$ or initiative? or prevent$ or program$ or reduc$ or strateg$ or treatment?) adj3 (alcohol$ or addict$ or cocaine or drug? or heroin or marijuana or narcotic$ or opioid?)).ti,kf. (88090)

36 ((effectiveness or improve$ or initiative? or prevent$ or program$ or reduc$ or strateg$ or treatment?) adj3 (alcohol$ or addict$ or cocaine or drug? or heroin or marijuana or narcotic$ or opioid?)).ab. (427373)

37 or/34-36 (583066)

38 ((overview$ or review or synthesis or summary or Cochrane or analysis) and (reviews or meta-analyses or articles or umbrella)).ti. (8903)

39 ((overview$ or reviews) and (systematic or cochrane)).ti. (8943)

40 (reviews adj2 meta).ab. (13288)

41 (reviews adj2 (published or quality or included or summar$)).ab. (13247)

42 (cochrane review* or systematic review*).ab. (214614)

43 (evidence and (reviews or meta-analyses)).ti. (1697)

44 or/39-43 (227862)

45 38 or 44 (231624)

46 animals/ not (humans/ and animals/) (5795904)

47 45 not 46 (230699)

48 10 and (16 or 23 or 33) and 37 and 47 (428)

49 remove duplicates from 48 (287)
