# Supplementary material for: The effectiveness of substance use interventions for homeless and vulnerably housed persons: A systematic review of systematic reviews on supervised consumption facilities, managed alcohol programs, and pharmacological agents for opioid use disorder
Source: PLoS One. 2020 Jan 16;15(1):e0227298. doi: 10.1371/journal.pone.0227298 (PMC6964917; doi:10.1371/journal.pone.0227298)
Supplement: S5 File — (PDF) [file pone.0227298.s005.pdf]

Appendix V: Table of Excluded Studies

Citation	Reason for Exclusion
Amato, L., Davoli, M., Perucci, C. A., Ferri, M., Faggiano, F., & Mattick, R. P. (2005). An overview of systematic reviews of the effectiveness of opiate maintenance therapies: available evidence to inform clinical practice and research. Journal of substance abuse treatment, 28(4), 321-329.	Wrong study design (review of reviews)
Amato, L., Davoli, M., Ferri, M., Gowing, L., & Perucci, C. A. (2004). Effectiveness of interventions on opiate withdrawal treatment: an overview of systematic reviews. Drug and alcohol dependence, 73(3), 219-226.	Wrong intervention (withdrawal)
Bart, G. (2012). Maintenance medication for opiate addiction: the foundation of recovery. Journal of addictive diseases, 31(3), 207-225.	Wrong study design
Canadian Nurses Association (2011). Harm reduction and currently illegal drugs: Implications for nursing policy, practice, education and research. Discussion Paper.	Wrong study design
Canadian Nurses Association (2017). Harm reduction & illicit substance use: Implications for Nursing.	Wrong study design
Chimbar, L., & Moleta, Y. (2018). Naloxone Effectiveness: A Systematic Review. Journal of addictions nursing, 29(3), 167-171.	Wrong study design, intervention
Crowley, D., & Van Hout, M.C. (2017). Effectiveness of pharmacotherapies in increasing treatment retention and reducing opioid overdose death in individuals recently released from prison: a systematic review. Heroin Addiction and Related Clinical Problems, 19(2).	Wrong population
Davids, E., & Gastpar, M. (2004). Buprenorphine in the treatment of opioid dependence. European Neuropsychopharmacology, 14(3), 209-216.	Wrong outcomes
Dennis, B., Bawor, M., Naji, L., Boner, A., Roshanov, P., Paul, J., ... & Plater, C. (2018). 48 Trials and tribulations of establishing treatment effectiveness in addiction research. Bmj Evidenced-based Medicine, 23(Suppl_1).	Wrong study design
Faggiano, F., Versino, E., Vigna-Taglianti, F., & Lemma, P. (2003).Methadone maintenance at different dosages for opioid dependance.The Cochrane Library, Issue 3.	Wrong intervention (dosing)
Gowing, Linda ; Farrell, Michael ; Ali, Robert ; White, Jason M. Alpha$_2$-adrenergic agonists for the management of opioid withdrawal The Cochrane database of systematic reviews, 03 May 2016 (5), pp.CD002024	Wrong intervention (withdrawal)
Hedrich, D., Alves, P., Farrell, M., Stöver, H., Møller, L., & Mayet, S. (2012). The effectiveness of opioid maintenance treatment in prison settings: a systematic review. Addiction, 107(3), 501-517.	Wrong population
Holloway, K. R., Bennett, T. H., & Farrington, D. P. (2006). The effectiveness of drug treatment programs in reducing criminal behavior: A meta-analysis. Psicothema, 18(3), 620-629.	Wrong outcomes
Jones, J. L., Mateus, C. F., Malcolm, R. J., Brady, K. T., & Back, S. E. (2018). Efficacy of ketamine in the treatment of substance use disorders: a systematic review. Frontiers in Psychiatry, 9.	Wrong intervention

Larney, S. (2010). Does opioid substitution treatment in prisons reduce injecting-related HIV risk behaviours? A systematic review. Addiction, 105(2), 216-223.	Wrong population
Maglione, M. A., Raaen, L., Chen, C., Azhar, G., Shahidinia, N., Shen, M., ... & Hempel, S. (2018). Effects of medication assisted treatment (MAT) for opioid use disorder on functional outcomes: A systematic review. Journal of substance abuse treatment, 89, 28-51.	Wrong outcomes
Mathew C, Mendonca O, Abdalla T, Stergiopoulos V, Bloch G,...Pottie K (2018). Interventions to improve physical health, mental health and social outcomes for homeless ad vulnerably housed populations. The Cochrane Collaboration.	Wrong study design
Mauger, S., Fraser, R., & Gill, K. (2014). Utilizing buprenorphine–naloxone to treat illicit and prescription-opioid dependence. Neuropsychiatric disease and treatment, 10, 587.	Wrong study design
May, T., Bennett, T., & Holloway, K. (2018). The impact of medically supervised injection centres on drug-related harms: A meta-analysis. International Journal of Drug Policy, 59, 98-107.	Retracted from publication
McAuley, A., Aucott, L., & Matheson, C. (2015). Exploring the life-saving potential of naloxone: a systematic review and descriptive meta-analysis of take home naloxone (THN) programmes for opioid users. International Journal of Drug Policy, 26(12), 1183-1188.	Wrong outcomes
McNeil, R., & Small, W. (2014). 'Safer environment interventions': A qualitative synthesis of the experiences and perceptions of people who inject drugs. Social Science & Medicine, 106, 151-158.	Wrong outcomes
Nielsen, S., MacDonald, T., & Johnson, J. L. (2018). Identifying and treating codeine dependence: a systematic review. Medical Journal of Australia, 208(10), 451-461.	Wrong outcomes
Poulin, C. (2006). Harm reduction policies and programs for youth. Canadian Centre on Substance Abuse.	Wrong study design
Pujol, C. N., Paasche, C., Laprevote, V., Trojak, B., Vidailhet, P., Bacon, E., & Lalanne, L. (2017). Cognitive effects of labeled addictolytic medications. Progress in Neuro-Psychopharmacology and Biological Psychiatry.	Wrong intervention
Rahimi-Movaghar, A., Amin-Esmaeili, M., Hefazi, M., & Yousefi-Nooraie, R. (2013). Pharmacological therapies for maintenance treatments of opium dependence. The Cochrane database of systematic reviews, (1), CD007775-CD007775.	Wrong intervention
Ramsperger E & Ramage K. (2017). A selective literature review on managed alcohol programs and indigenous healing methodologies. Aboriginal Standing Committee on Housing and Homelessness	Wrong population
Rapid Response Service. Rapid Response: What is the effectiveness of supervised injection services? Toronto, ON: Ontario HIV Treatment Network; May 2014.	Wrong study design
Rayburn, W. F., & Bogenschutz, M. P. (2004). Pharmacotherapy for pregnant women with addictions. American Journal of Obstetrics and Gynecology, 191(6), 1885-1897.	Wrong study design
Reed, M. (2008). Wet Shelters: The benefits and risks associated with alcohol-administering homeless shelters. Ministry of Labour and Citizens Services, British Columbia, Canada.	Wrong study design
Snooks, H., Russell, D., Brown, C., Nair, A., Moore, C., Lewis, A., ... & Griffith-Noble, F. (2011). 015	Wrong study

How can we prevent overdoses and what works? a systematic review of interventions for non fatal poisonings. Emergency Medicine Journal, 28(3), e1-e1.	design
Sokol, R., LaVertu, A. E., Morrill, D., Albanese, C., & Schuman-Olivier, Z. (2018). Group-based treatment of opioid use disorder with buprenorphine: A systematic review. Journal of substance abuse treatment, 84, 78-87.	Wrong intervention
Tsai, L. C., & Doan, T. J. (2016). Breastfeeding among mothers on opioid maintenance treatment: a literature review. Journal of Human Lactation, 32(3), 521-529.	Wrong outcomes
