# Supplementary material for: The effectiveness of substance use interventions for homeless and vulnerably housed persons: A systematic review of systematic reviews on supervised consumption facilities, managed alcohol programs, and pharmacological agents for opioid use disorder
Source: PLoS One. 2020 Jan 16;15(1):e0227298. doi: 10.1371/journal.pone.0227298 (PMC6964917; doi:10.1371/journal.pone.0227298)
Supplement: S4 File — (PDF) [file pone.0227298.s004.pdf]

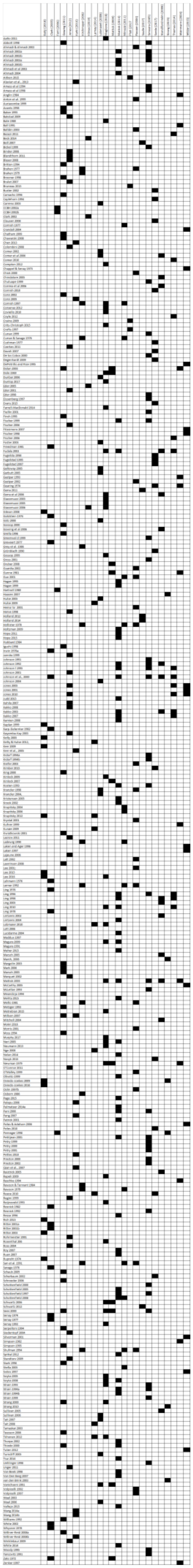

	Kennedy (2017)	Potier (2014)
Andresen & Boyd 2010	■	■
Andresen & Jozaghi 2012	■	
Baars 2010	■	
Bayoumi & Zaric 2008	■	■
Bravo 2009	■	■
Cruz 2007		■
DeBeck 2008		■
DeBeck 2011a	■	■
DeBeck 2011b		■
Donnelly & Mahoney 2013	■	
Dubois-Arber 2008		■
Fairbairn 2008		■
Fast 2008		■
Fitzgerald 2010	■	■
Freeman 2005	■	■
Fry 2002		■
Green 2004		■
Hadland 2014		■
Hedrich 2004	■	
Jozaghi & Andresen 2013		■
Jozaghi 2013		■
Jozaghi 2014	■	
Kerr 2005c	■	■
Kerr 2006a	■	■
Kerr 2006b		■
Kerr 2007a		■
Kerr 2007b	■	■
Kimber & Dolan 2007		■
Kimber 2003		■
Kimber 2008a		■
Kimber 2008b	■	■
Kinnard 2014	■	
Kral 2010		■
Lloyd-Smith 2008	■	■
Lloyd-Smith 2009		■
Lloyd-Smith 2010	■	■
Lloyd-Smith 2012	■	
Lysyshyn 2017	■	
Marshall 2009	■	■
Marshall 2011	■	■
McKnight 2007		■
McNeil 2013		■
Milloy & Wood 2009		■
Milloy 2008a	■	■
Milloy 2008b	■	■
Milloy 2009	■	■
Milloy 2010	■	■
Navarro & Leonard 2004		■
NCHECR 2007	■	
O'Shea 2007		■
Petrar 2007		■
Philbin 2009		■
Pinkerton 2010	■	■
Pinkerton 2011	■	■
Poschadel 2003	■	
Reddon 2011		■
Richardson 2008	■	■
Salmon 2007	■	■
Salmon 2009a		■
Salmon 2009b		■
Salmon 2010	■	■
Scherbaum 2010	■	
Skelton 2016	■	
Small 2008		■
Small 2009		■
Small 2011a		■
Small 2011b		■
Small 2012		■
Stoltz 2007a		■
Stoltz 2007b	■	■
Thein 2005	■	■
Toth 2016	■	
Tyndall 2006a		■
Tyndall 2006b		■
Van Beek & Gilmour 2000		■
Van Beek 2004	■	■
Vecino 2013	■	
Watson 2012		■
Wood 2003		■
Wood 2004	■	■
Wood 2005a		■
Wood 2005b	■	■
Wood 2005c		■
Wood 2005d		■
Wood 2006a	■	■
Wood 2006c		■
Wood 2006d	■	■
Wood 2007	■	■
Wood 2008	■	■
Zurhold 2003	■	
